# School-Based Nursing Interventions for Preventing Bullying and Reducing Its Incidence on Students: A Scoping Review

**DOI:** 10.3390/ijerph20021577

**Published:** 2023-01-15

**Authors:** Iyus Yosep, Rohman Hikmat, Ai Mardhiyah

**Affiliations:** 1 Department of Mental Health, Faculty of Nursing, Universitas Padjadjaran, Bandung 40132, Indonesia; 2 Faculty of Nursing, Universitas Padjadjaran, Bandung 40132, Indonesia; 3 Department of Pediatric Nursing, Faculty of Nursing, Universitas Padjadjaran, Bandung 40132, Indonesia

**Keywords:** bullying, school based nursing interventions, students

## Abstract

Incidents of bullying have increased on students in schools. This has a negative impact such as mental health problems and risk of suicide. Interventions related to bullying are still focused on being carried out independently. Meanwhile, collaborative interventions between nurses and the school are needed to acquire maximum results in preventing and reducing the incidence of bullying. The purpose of this study is to describe school-based nursing interventions in preventing and reducing the incidence of bullying among students at school. This study used scoping review with a search strategy using the PRISMA Extension for Scoping Review process to find the articles. The PubMed, CINAHL, and Scopus electronic databases were searched. We found 12 articles from 594 articles in 3 databases which discussed nursing interventions based on school to prevent and reduce incidence of bullying in students. The studies included are design randomized control trials and quasi experiments. The samples with the range of 40–7121 respondents. We classified the school-based nursing interventions being three methods, there are Game programs, physical activity programs, training programs, and peer-group programs. The content of interventions are psychoeducation, empathy training, counseling, and self-management. This study shows that school-based nursing interventions can be an option in preventing and reducing the incidence of bullying among students at school.

## 1. Introduction

Prevalence of bullying on students increased every year. A survey conducted by Latitude News of 40 countries revealed cases of bullying and made Indonesia in second place after Japan with frequent cases of bullying, followed by Canada, South Korea, US and Finland [1]. In 2016, data from UNICEF showed that 50% from 1000 adolescents in Indonesia in the age range of 13 to 15 years have experienced acts of bullying [2]. From data on bullying cases in America reported by the Josephson Institute of Ethics which conducted survey of 43,000 adolescents, the results were 47% of adolescents aged 15–18 years had experienced bullying and 50% of adolescents had teased, disturbed, and ridiculed [3]. In addition to America, in Japan, it has been found that 40% of 5000 students reported being victims of bullying, they reported having been ridiculed, ridiculed, ostracized, beaten, kicked at least once a week [4]. Another study showed that 30% of 1000 students in Africa have experienced bullying [5].

Bullying is when someone repeatedly and on purpose says or does mean or hurtful things to another person who has a hard time defending himself or herself [6]. Other definition said that bullying is any form of violence that is carried out intentionally by one person or group of people towards another person, with the aim of causing harm and is carried out continuously [7]. Bullying that occurs in students takes the form of ridiculing, spreading gossip, giving nicknames, hurting verbally or in writing, isolating, intimidating, and even physically attacking [8]. Meanwhile, according to other studies, forms of bullying behavior such as insulting, shouting, giving inappropriate nicknames, slandering, slandering, and embarrassing in public are forms of verbal bullying behavior [9]. Forms of physical bullying behavior include pushing, hitting, punching, kicking, pinching, and grabbing [10]. Bullying in the form of threats is such as making gestures or writing threatening notes [11]. Next, cyberbullying is sending messages or images that embarrass other individuals on social media [12].

The negative impact of bullying on students was negative emotions such as anger, sadness, and revenge, which causes students to withdraw from the environment. In addition, the victim of cyberbullying have low self-esteem and be more sensitive of environmental responses [13]. The worst impact of bullying on students are psychological problems such as depression, leading to the idea of committing suicide [12,14]. The perpetrators of bullying also acquire negative impacts such as losing their future, and being subject to legal sanctions [15]. Students who are victims of bullying have problems with difficulties in building interpersonal relationships with other people and rarely come to school [16]. Victims of bullying have difficulty communicating and concentrating in learning so that this affects physical and mental health both in the short and long term [17]. The worst impact that occurs on victims of bullying is the risk of suicide due to bullying experienced [18].

Nurses as professional health workers can collaborate with schools. Nurses can carry out their roles as educators and advocates for children, youth, parents, teachers, and other communities related to actions and prevention efforts, as well as efforts to overcome bullying [19]. Nurses have role as counselors, can work together in developing educational programs and conducting interventions related to bullying as a prevention effort [15].

Collaboration between nurses and the school is important to implement for preventing and reducing the impact of bullying on students at school. The results of previous studies showed that school-based nursing interventions can effectively raise student awareness about bullying [20]. Other studies also show that collaborative interventions between teachers and nurses are rarely used to deal with students who experience bullying [21]. However, several studies show that independent nursing interventions take 2 years to restore the impact of bullying on students. Another study showed that bullying prevention interventions carried out by teachers in schools did not significantly reduce the incidence of bullying. Many nursing interventions to prevent bullying are carried out independently so they are not integrated with one another [22]. There is a problem gap, namely the lack of collaboration between nurses and teachers in preventing and reducing the negative effects of bullying on students.

School-based interventions involving nurses are important to prevent and reduce the impact of bullying on students at school. However, nurses and schools are still not exposed to information about collaborative interventions that can be carried out. Therefore, a scoping review is needed to describe school-based interventions carried out with nurses to prevent and reduce the impact of bullying on students at school.

## 2. Methods

### 2.1. Design

The design used in this study was scoping review. The aim of this method for discussing and exploring interesting topics and issues that are currently developing [23]. Scoping review was chosen by the authors because it has a broad conceptual range so that it can answer the research objectives that have been set [24]. The stages used in this study were determining research questions, determining keywords as an article search strategy, searching articles based on predetermined keywords, analyzing and compiling the results of article reviews, and preparing a report on study results [25]. This study used PRISMA Extension for Scoping Reviews (PRISMA-ScR) to identify various topics that address school-based nursing interventions that can be implemented to prevent and reduce the impact of bullying on students in schools.

### 2.2. Search Methods

The articles used in this scoping review were obtained from the PubMed, CINAHL, and Scopus databases. The keywords used are: “school-based intervention OR school-based program” AND “nursing intervention OR nursing care” AND “student” AND “bullying” AND “prevent OR reduce impact”. The research question is what are the method of school-based nursing interventions to prevent and reduce the incidence of bullying among students at school?

### 2.3. Inclusion and Exclusion Criteria

**Figure 1:**
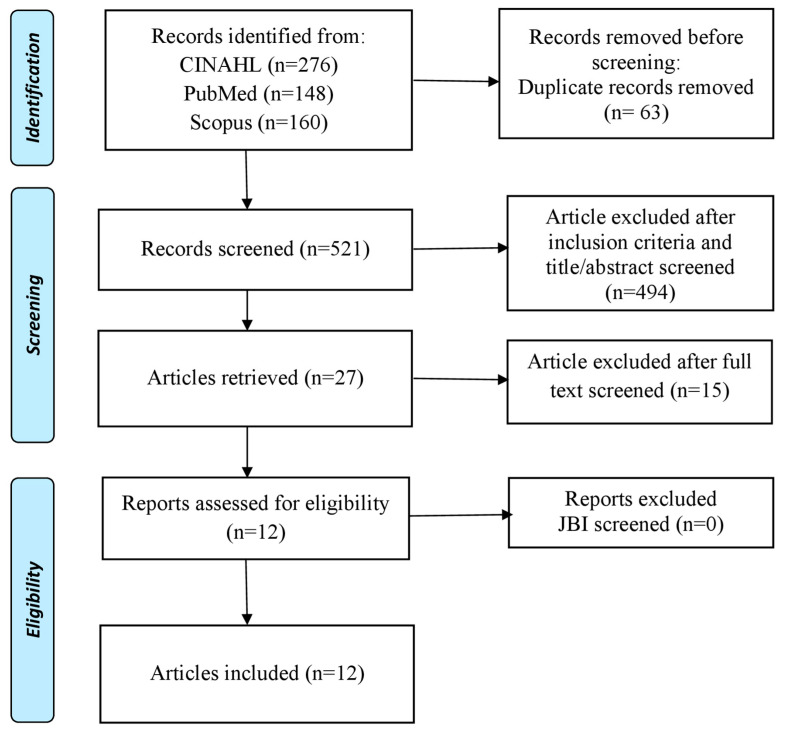
PRISMA Flow Diagram.

This study used the PRISMA Extension for Scoping Review (PRISM-ScR) to identify the types of interventions that can be carried out to prevent and reduce the incidence of bullying among students using school-based nursing interventions (Figure 1). This study has inclusion and exclusion criteria in selecting articles. The inclusion criteria in this study were that the sample consisted of students, primary research, school-based nursing interventions, randomized control trial or quasi-experimental research design, using English, and the time of publication being the last 10 years (2013–2022). The exclusion criteria in this study were that the intervention did not involve nurses, the intervention was carried out independently, and the sample was not students.

### 2.4. Data Extraction

Data extractions were carried out by the authors to make it easier to analyze the results of the articles that have been reviewed. The data extraction process was carried out by creating a manual table. The aspects written in the extraction table were the author, year, country, research design, population and sample, procedures, interventions, and results of the study. The purpose of the authors to make an extraction table is to simplify the process of analyzing the results of the review to be used as a description of the results based on the classification of school-based nursing interventions.

### 2.5. Quality Appraisal

The authors evaluate the quality of the article used The Joanna Brigs Institute (JBI). The JBI assessment method is an instrument for measuring the quality level of articles based on the type of research. Assessment used the scoring method for each statement of the instrument and is given a value of yes, no, unclear, and not applicable. The score “yes” is given a value of 1, while the other scores are given a value of zero. The standard value of the article based on the JBI assessment for use in this study is above 75% based on the criteria and topic relevance.

### 2.6. Data Analysis

The articles were obtained by the authors based on the results of the selection of inclusion and exclusion criteria. Next the quality of the articles was assessed using the JBI method. The articles were then read in full and analyzed based on the extraction table by all authors. Next the authors performed data analysis with a descriptive approach. The results of data analysis are reported by classifying the results of school-based nursing interventions to prevent and reduce the negative impact of bullying on students at school.

## 3. Results

The number of articles obtained from the search database is 594 articles. After duplicating the collected articles, 521 articles were obtained. Furthermore, after elimination based on the inclusion criteria and after checking the title and abstract, 37 articles were found. We read full text and selection the articles based on exclusion criteria and relevance of topic based on purpose the study, and we found 12 articles to analyzed in this study. Authors used the JBI Critical Appraisal Tool assessment method to analyze the quality of articles and all authors gave the standard score of analyzed articles if it was above 75%, based on criteria and topic relevance (Table 1).

The authors wrote the review of articles in manual table to show the result of the study. The aspect in the table extraction is references, purpose, country, method, sample, intervention, and results. The analysis of the article in this study are presented as follow (Table 2).

There were 12 articles which discussed about school-based nursing interventions to prevent and reduce the negative impact of bullying on students in school. Most nursing interventions are carried out in developed countries (Australia, United Kingdom, United States, Spain, London, and Sweden). The range of samples is 40–7121 respondents. Most of the respondents came from London.

This study showed that nurses and teachers need to work together for preventing and reducing the incidence of bullying in students. Nurses have the role to provide comprehensive nursing care from biological, psychological, sociological, and spiritual aspects. One of the roles of community nurses in the mental health aspect is school nurses who provide comprehensive school nursing care to improve the health of students and school employees including physical and psychological health. The role of nurses as educators to increase knowledge of bullying on students and increase student awareness of bullying incidents. Nurses also have a role as advocates to collaborate with teachers regarding policies in schools regarding bullying. In addition, nurses have a role as facilitators with teachers to guide and supervise activities carried out to prevent and reduce the impact of bullying on students. The role of the counselor as a nurse is carried out to overcome the impact of trauma experienced by students at school due to bullying in collaboration with teachers. Teachers as partners in schools form learning curricula to prevent bullying. The role of teachers and nurses is an important role to prevent and reduce the incidence of bullying in schools.

The Authors have read and analyzed 12 articles, and we are classified the intervention in four methods, there were Game program, physical activity program, training program, and peer-group program. The explanation of each school-based nursing intervention method is as follows.

### 3.1. Game Programs

The game program is a method of school-based nursing intervention by playing games with students at school. Collaborative Board Game is carried out for 5 weeks (1 h/session) [26]. The nurse has a role as a facilitator to guide the game to students. The game starts with the facilitator give the video instructions about game, then the participants play the game. During breaks, participants reflect the game with facilitator regarding the knowledge, attitudes, and empathy for bullying for each player. This game can foster empathy and self-confidence in students when bullied.

The SEHER program with games is a multi-component intervention that emphasizes the importance of a positive school climate, namely a supportive relationship between school members [33]. Nurses have a role to make a module about games and evaluate the results of games played by students online. Participants take part in a series of activities starting from psychoeducation and games guided through videos instruction. The results of the intervention showed that there was a decrease in bullying risk behavior in the school community, and increased problem-solving skills among adolescents.

### 3.2. Physical Activity Program

Physical activity carried out together can increase a sense of concern for friends, so as to prevent bullying from happening to students. One method that can be carried out is Martial Arts Based Intervention carried out for 10 weeks for 50 min per week [27]. The intervention began with the provision of psychoeducation guided by the facilitator regarding goal setting, self-concept and self-esteem, courage, resilience, intimidation and peer pressure, self-care and concern for others, values, and optimism and hope. Nurses collaborate with physical activity therapists to evaluate the results of activity therapy performed by students. Nurses have a role in monitoring and evaluating physical activity therapy. Next the participants warm up (jogging, push-up, and sit ups), then the participants do stretching namely hamstring stretching, triceps stretching, stretching, and standing quad stretching, in the final stage the participants will do martial arts exercises including stances, blocks, punching, and kicking. This activity is carried out in groups, the facilitator also teaches about the meaning of caring for friends during physical activity. This has a positive impact on students’ empathy so as to reduce bullying to their friends. The results of the study showed that the participants experienced an increase in mental health after the intervention.

ACT OUT! Social Issue Theater Program is an education that is packaged through drama [29]. Participants perform an interactive, semi-improvised psychodrama performance for one hour. Facilitators give guidelines about the interventions. The activity includes 3 vignettes paired with moderated discussion between audience and actors, and the last will remain in character throughout the intervention. The nurse’s role is to oversee the process of making drama scripts and evaluate the results of the drama displayed by students. The drama played by students is about bullying and the impact of bullying. Providing a moral message through this drama is an effort to prevent bullying from happening to students. The results of the intervention showed that there was a significant effect in increasing resilience and reducing mental health problems in students who experienced bullying.

### 3.3. Training Programs

Training is an activity that is mostly carried out in school-based nursing interventions. The method used is in the form of Web-Enabled, School-Based, Preventive Intervention carried out for 10 sessions of 40 min/session [28]. Nurses as educators have a role in providing education to students and teachers about how to deal with the negative effects of bullying. This educational program has a purpose for improving knowledge of bullying, promoting respect for diversity, empathy, emotion management, and the development of social skills. The results of the intervention showed that there was a reduction in the incidence of bullying among students at school.

Multicomponent secondary school health promotion intervention was carried out for 4 weeks [30]. Nurses provide education about bullying to increase student understanding so as not to bully and also as counselors to overcome the traumatic effects of bullying. Health education activities include material on bullying, the impact of bullying, empathy, emotional management, and a culture of mutual respect. Next the participants were asked to make a project related to preventing bullying in the school environment. After the intervention, there was a reduction in the incidence of bullying in schools.

SEHER program with education is an activity carried out for 10 weeks. This activity begins with holding workshops with all school officials, parents, and students regarding the activities to be carried out [31]. Nurses have a role as educators and mentors in increasing the knowledge of students, parents and teachers about bullying and how to deal with trauma caused by bullying. Activities consist of health education, peer groups, improving learning skills such as time management, learning styles, note-taking, reading comprehension, memorization techniques, and concentration techniques. The results of the intervention showed an increase in empathy for students at school.

Teaching Recovery Techniques (TRT) is a training activity conducted for 7 weeks [34]. This activity focuses on psychoeducation, intrusion, arousal, avoidance, and coping strategies. Nurses provide education to students at school about bullying and provide consulting services to victims of bullying and improve students’ mental health. Each session involves skills training in empathy and problem solving. In the last session, the participants will consolidate learning and talk about their experiences. The results of the intervention showed a significant reduction in the incidence of bullying at school.

Pragmatic school-based universal intervention is a training activity given to students for 8 weeks [35]. Nurses become trainers to improve skill of students such as empathy and problem solving, and also as counselors to students who need further therapy to improve their mental health. Training activities include increasing empathy, problem solving, and interaction with others. The results of the intervention showed a de-crease in the incidence of bullying among students at school.

### 3.4. Peer-Group Programs

Group activities are important to increase the sense of belonging to people. Learning Together intervention is a peer-group activity that discusses learning activities and also counseling on bullying issues [37]. The nurse has a role as facilitator to guide the discussion process in the group so that it is in accordance with the objectives of the intervention. Activities carried out for 10 weeks. This activity carried out for 6 weeks with significant results in reducing the incidence of bullying. Participants were also asked to present a drama about bullying [32]. The results of the intervention showed a reduction in the incidence of bullying at school among students.

The Tutoría Entre Iguales Program (school-based intervention of peer-tutoring) is a group peer-support activity guided by a facilitator for 6 weeks [36]. The nurses have a role as a facilitator to guide group discussions and as a counselor if there are students who need psychological support. This activity discusses the problems faced by students to deal with bullying. Each participant was provided with positive activities in terms of understanding and empathy for others.

## 4. Discussion

The results of this scoping review showed that school-based nursing interventions can prevent and reduce the incidence of bullying among students at school. The school-based nursing intervention methods used are game programs, physical activity programs, training programs, and peer-group programs. The activities carried out from each intervention were psychoeducation, empathy training, drama making, and peer-group.

This study showed that bullying often occurs in adolescent students. Previous study have shown that bullying is more common in high school students than university students [38]. Previous studies also show that 30% of 500 high school students aged 12–18 have experienced bullying at school [39]. This happens because adolescents are in a phase of searching for identity and unstable emotions, causing difficulties for adolescents in accepting differences between adolescents [40]. In line with previous studies which showed that unstable emotions in adolescents lead to uncontrolled behavior and speech of adolescents towards other people [41,42].

Implementation of school-based nursing interventions is carried out in a range of 5–10 weeks. This was adjusted to the characteristics of the victims and the participants’ previous knowledge regarding bullying. Previous studies have shown that preventive interventions effective to reduce bullying behavior in students take 8 weeks [43,44]. Bullying behavior is also still considered as something that is usually carried out by students, so building awareness related to bullying takes 2 months [45]. Another study also showed that nursing interventions to reduce the impact of bullying take 12 weeks for victims to return to their normal activities because victims have difficulty coming to terms with the traumatic experiences they have experienced [46,47]. This showed that the duration of the intervention varies according to the severity of the bullying experienced by the victim.

This study showed that incidence of bullying happened both in several developing and industrial countries. The number of incidents of bullying does not depend on the progress of a country [48]. Previous studies have shown that bullying tends to occur in developed countries [39,49]. Bullying also occurs frequently in developing countries, this is in line with previous studies which show that the prevalence of bullying has increased, one of which is in Indonesia [50].

Efforts to prevent and overcome school-based bullying can be carried out through the Game program. Games therapy as a medium for bullying prevention are carried out by increasing student awareness regarding bullying and its effects [51]. Nurses have a role for facilitating and guiding the course of games related to bullying [52]. Nurses can work together with teachers in making games to increase empathy between students so as to prevent bullying. Previous studies have shown that games about bullying will increase students’ knowledge and awareness regarding bullying [51,53]. Bullying occurs due to an individual’s lack of awareness and empathy for other people. School-based nursing interventions can be an option in reducing the incidence of bullying in students.

Bullying occurs because of a lack of student productivity in carrying out activities. School-based nursing interventions can be carried out to increase student productivity. Activities carried out in the form of theater exercises, physical activity training, and sports [50]. The nurse’s role is to provide advice about activities carried out by students and supervise student activities based on the stage of student development by working with the teacher. These positive activities will increase student productivity by utilizing the best possible time to do positive things. Physical activity carried out in groups can increase empathy. Previous studies have shown that physical activity can reduce students’ desire to bully [54,55]. In addition, physical activity can also make students think positively so that they can carry out positive activities.

Incidents of bullying in students occur because of a lack of empathy for other students. Victims of bullying also find it difficult to come to terms with traumatic experiences because they have un-adaptive coping. Nurses and teachers have a role in improving students’ adaptive coping in response to stress. Teachers and nurses can carry out counseling to students or even form peer-support groups between students. A program is needed to train students’ abilities to improve coping, empathy, and problem solving [56]. Adaptive coping can make students able to make peace with the problems they face [57]. This is in line with previous studies which show that nursing interventions with training methods can improve students’ adaptive coping [12,58]. In addition to coping, empathy and problem solving skills are also needed to come to terms with the traumatic experience of bullying. Victims of bullying with good problem-solving skills will seek solutions to the stressors that arise as a result of bullying [59]. This is supported by previous studies which show that victims of bullying need problem-solving exercises to find solutions to the problems they face [60].

Social support is an important thing for victims of bullying to have. The impact experienced by victims of bullying is social isolation and feeling lonely. The existence of social support through peer support groups can increase the confidence of victims of bullying [61,62]. Schools have an important role to play in forming peer-groups accompanied by facilitators in preventing and reducing the impact of bullying [63]. The nurse has a role as a facilitator to monitor and guide the implementation of the peer-group carried out by students. Students will discuss and carry out activities to prevent bullying from happening to students under the supervision of the nurses. Nurses also provide directions and guidance modules in conducting peer support groups [64]. Peer-group intervention can also increase self-confidence so that it can reduce feelings of loneliness and social isolation in victims [65].

School-based nursing interventions have an important role in preventing and reducing the impact of bullying on students. Nurses can provide comprehensive nursing care in collaboration with schools as educators, facilitators and counselors. Each program can be implemented to foster student self-confidence and awareness in students so they do not bully other students. In addition, interventions carried out by nurses can also reduce the impact of bullying such as stress, depression, and social isolation.

### Limitations

The limitations in this study are the limited study design, namely randomized control trials and quasi experiments. Although the authors aim to obtain interventions that are accurate in preventing and reducing the impact of bullying, this study cannot discuss the results of other studies with more diverse designs. The time of publication of the articles used as literature to be reviewed in this study is also limited to the last 10 years, so the authors cannot comprehensively discuss and compare interventions carried out in the present and in the past. In addition, the database in this study is limited to 3 databases, but this study cannot find other data to be a comparison of the study results obtained.

## 5. Conclusions

The authors found 12 articles discussed about school-based nursing interventions for preventing and reducing the negative impact of bullying on students. This study showed that intervention carried out in collaboration between health workers and the school can optimize in reducing incidence of bullying. There are three methods used for providing school-based nursing interventions are through Game programs, physical activity programs, training programs, and peer-group programs. Activities undertaken include psychoeducation, empathy training, counseling, and self-management. Interventions also require a facilitator to support and accompany participants in carrying out each activity that has been determined. The implication of this study is being a basis for nurses to provide interventions to prevent and reduce the impact of bullying that occurs on students. Another implication that can be given is the existence of policy advice for schools and health services for reducing the negative impact and incidence of bullying. The suggestion for future research is needed the randomized control trial design to analyze the effectiveness of nursing interventions carried out with a family approach in reducing the negative effects of bullying.

## Figures and Tables

**Table 1:** JBI Critical Appraisal Tool.

Author, Pubslished Year	Title	JBI Critical Appraisal Tool	Study Design
(Nieh & Wu, 2018) [25]	Effects of a collaborative board game on bullying intervention: a group-randomized controlled trial	84.6% (11/13)	RCT
(Moore, Woodcock & Dudley, 2018) [26]	Developing wellbeing through a randomised controlled trial of a martial arts based intervention: an alternative to the anti-bullying approach	92.3% (12/13)	RCT
(Díaz-Caneja et al., 2021) [27]	Efficacy of a web-enabled, school-based, preventative intervention to reduce bullying and improve mental health in children and adolescents: study protocol for a cluster randomized controlled trial	76.9% (10/13)	RCT
(Agley et al., 2021) [28]	Effects of ACT Out! social issue theater on social-emotional competence and bullying in youth and adolescents: cluster randomized controlled trial	76.9% (10/13)	RCT
(Shinde et al., 2020) [29]	A multicomponent secondary school health promotion intervention and adolescent health: an extension of the SEHER cluster randomised controlled trial in Bihar, India	92.3% (12/13)	RCT
(Shinde et al., 2018) [30]	Promoting school climate and health outcomes with the SEHER multi-component secondary school intervention in Bihar, India: a cluster-randomised controlled trial	84.6% (11/13)	RCT
(Christopher Bonell et al., 2020) [31]	Broader impacts of an intervention to transform school environments on student behaviour and school functioning: post hoc analyses from the INCLUSIVE cluster randomised controlled trial	84.6% (11/13)	RCT
(Singla, Shinde, Patton & Patel, 2021) [32]	The mediating effect of school climate on adolescent mental health: findings from a randomized controlled trial of a school-wide intervention	92.3% (12/13)	RCT
(Durbeej et al., 2021) [33]	Evaluation of a school-based intervention to promote mental health of refugee youth in Sweden (The RefugeesWellSchool Trial): study protocol for a cluster randomized controlled trial	76.9% (10/13)	RCT
(Dray et al., 2017) [34]	Effectiveness of a pragmatic school-based universal intervention targeting student resilience protective factors in reducing mental health problems in adolescents	76.9% (10/13)	RCT
(Ferrer-Cascales et al., 2019) [35]	Effects of the learning together intervention on bullying and aggression in english secondary schools (inclusive): a cluster randomised controlled trial	90.9% (10/11)	Quasy experimental
(Chris Bonell et al., 2018) [36]	Evidence-based intervention against bullying and cyberbullying: Evaluation of the NoTrap! program in two independent trials	92.3% (12/13)	RCT

**Table 2:** Extraction Data.

No	Reference	Purpose	Country	Method	Sample	Intervention	Result
1.	[26]	Effects of the Galaxy Rescuers game to reduce bullying	Taiwan	RCT	328 students ages of 10–17	Collaborative Board Game	Significantly improve awareness of bullying on students and reduce incidence of bullying
2.	[27]	Effects of a martial arts based to reduce negative impact of bullying by improve resilience	Australia	RCT	283 students ages of 14–18	Martial Arts Based Intervention	Effectively reduce negative impact of bullying
3.	[28]	Effects interventions on mental health and quality of life	Australia	RCT	200 students ages of 12–18	Web-Enabled, School-Based, Preventative Intervention	Effectively improve mental health of bullying victims
4.	[29]	Effect brief psychodramatic intervention to reduce bullying behavior in students	United States	RCT	80 students ages of 10–18	ACT OUT! Social Issue Theater Program	Significantly reduce bullying behavior on students
5.	[30]	Effects of the intervention to improve resilience and wellbeing of students	India	RCT	75 students ages of 13–18	Multicomponent secondary school health promotion intervention	Effectively improve resilience and well-being of students
6.	[31]	Effect interventions on improve adolescent health and wellbeing	India	RCT	75 students ages of 11–18	SEHER program	Significantly improve adolescent health and wellbeing as victims of bullying
7.	[32]	To tested the Learning Together intervention to developing social and emotional skills	United Kingdom	RCT	40 students ages of 12–18	Learning Together intervention	Significantly improve developing social and emotional skills on students as victims of bullying
8.	[33]	Effect the interventions to improve mental health	India	RCT	5539 students ages of 10–18	SEHER Program	Significantly improve mental health on students as victims of bullying
9.	[34]	Effect the interventions to reduce PTSD	Sweden	RCT	700 students ages of 12–17	Teaching Recovery Techniques (TRT)	Effectively reduce symptoms of PTSD on students as victims of bullying
10.	[35]	Effect of interventions on improve mental health	Australia	RCT	315 students ages of 13–17	Pragmatic school-based universal intervention	Significantly improve mental health by resilience
11.	[36]	Effect the interventions to reduce incidence of bullying	Spain	Quasy experimental	2057 students ages of 10–18	Tutoría Entre Iguales Program	Significantly reduce incidence of bullying
12.	[37]	Effect the intervention in developing students’ social and emotional skills to prevent bullying	London	RCT	7121 students ages of 11–18	Learning Together intervention	Effectively prevent bullying by developing students’ social and emotional skills

## Data Availability

Not applicable.
